# Sepsis biomarkers and diagnostic tools with a focus on machine learning

**DOI:** 10.1016/j.ebiom.2022.104394

**Published:** 2022-12-02

**Authors:** Matthieu Komorowski, Ashleigh Green, Kate C. Tatham, Christopher Seymour, David Antcliffe

**Affiliations:** a Division of Anaesthetics, Pain Medicine, and Intensive Care, Department of Surgery and Cancer, Faculty of Medicine, Imperial College London, London, SW7 2AZ, United Kingdom; b Anaesthetics, Perioperative Medicine and Pain Department, Royal Marsden NHS Foundation Trust, 203 Fulham Rd, London, SW3 6JJ, United Kingdom; c Department of Critical Care Medicine, School of Medicine, University of Pittsburgh, Pittsburgh, PA, USA

**Keywords:** Sepsis, Biomarkers, Machine learning, Phenotypes, Clustering, Precision medicine

## Abstract

Over the last years, there have been advances in the use of data-driven techniques to improve the definition, early recognition, subtypes characterisation, prognostication and treatment personalisation of sepsis. Some of those involve the discovery or evaluation of biomarkers or digital signatures of sepsis or sepsis sub-phenotypes. It is hoped that their identification may improve timeliness and accuracy of diagnosis, suggest physiological pathways and therapeutic targets, inform targeted recruitment into clinical trials, and optimise clinical management. Given the complexities of the sepsis response, panels of biomarkers or models combining biomarkers and clinical data are necessary, as well as specific data analysis methods, which broadly fall under the scope of machine learning. This narrative review gives a brief overview of the main machine learning techniques (mainly in the realms of supervised and unsupervised methods) and published applications that have been used to create sepsis diagnostic tools and identify biomarkers.

## Introduction

Sepsis is an abnormal systemic reaction to an infection, representing a pattern of response by the immune system to infectious agents. Often, a hyper-inflammatory response is followed by an immunosuppressive phase during which secondary infections and multiple organ dysfunction can occur.^1^^,^^2^

Sepsis, as currently defined, includes a wide variety of heterogeneous non-specific clinical pictures. As Liu puts it: “sepsis is not a monolithic disease, but a loose collection of symptoms with diverse outcomes”.^3^ It has long been clear that the “definitions of sepsis may be too broad and common to heterogeneous groups of patients who do not necessarily have the same disorder”.^4^ The consequence of the challenge of characterising sepsis is that it complicates its early identification and the successful application of personalised interventions (such as steroids, immunotherapy or haemodynamic optimisation).^5^

Over the last decades, there have been a number of advances in the use of data-driven techniques to improve on the definition, early recognition, subtypes characterisation, and personalisation of management. Some of those broadly involve the discovery or assessment of biomarkers or digital signatures of sepsis or sepsis phenotypes. It is hoped that the identification of sepsis biomarkers and/or subclasses may improve timeliness and accuracy of diagnosis, suggest physiological pathways and therapeutic targets, inform targeted recruitment into clinical trials, and optimise clinical management.^5^

Here, we use the term biomarker loosely, including any molecule (inclusive of routinely measured lab tests) measured in the blood that have the potential to help with the above mentioned objectives. Given the complexities of the sepsis response, panels of biomarkers or models combining biomarkers and clinical data are necessary.^2^^,^^5^ Therefore, we also analysed the literature combining biomarkers with other patient data including demographics, vital signs, etc. Due to the complexity, high dimensionality and/or large size of the datasets involved, specific data analysis tools –loosely termed machine learning– become required. A wide range of sepsis biomarkers has been described, including fluid phase pattern recognition molecules (PRMs), cytokines, chemokines, damage-associated molecular patterns (DAMPs), non-coding RNAs, microRNAs, cell membrane receptors, cell proteins, metabolites, soluble receptors and complement system components.^2^^,^^6^ These biomarkers can be classified in their various “omics” categories: (epi)genomics (the study of the genome and its supporting structures), transcriptomics (RNA), proteomics (proteins including cytokines), and metabolomics (metabolites).^7^ Among those, we excluded most of the research that did not involve a machine learning component, since it has been exhaustively reviewed recently elsewhere.^2^^,^^6^^,^^7^

Machine learning is a class of mathematical methods that attempt to generate knowledge and insight from large datasets. We have included a section introducing the most common machine learning methods in this review. In a nutshell, supervised learning enabled the development of sepsis prediction algorithms, whilst unsupervised learning has been applied to highlight the underlying structure or to unearth hidden patterns in high-dimensional datasets, which is particularly appealing given the above-mentioned issues around sepsis characterisation. The vast majority of applications on sepsis diagnosis and prognostication rely on supervised learning, whilst unsupervised learning is used to define phenotypes (and their downstream applications in decision support).

Due to the vastness of the existing literature and the heterogeneity of the topics included in this review, we chose to conduct a narrative review, which are suitable in situations when there are disparate interventions or when there is dissimilarity of outcome measures and follow-up times in the analysed material.^8^ This narrative review will give a brief overview of the main machine learning techniques that have been used to create sepsis diagnostic tools and identify biomarkers, and survey the literature on these applications. We structured the review based on the purpose of the models (diagnosis, prognosis, or phenotyping) and the nature of the input data: routine (e.g. clinical data and laboratory tests) or non-routine data (gene expression, metabolomics, cytokines, etc.).

## Overview of relevant machine learning techniques

**Fig. 1:**
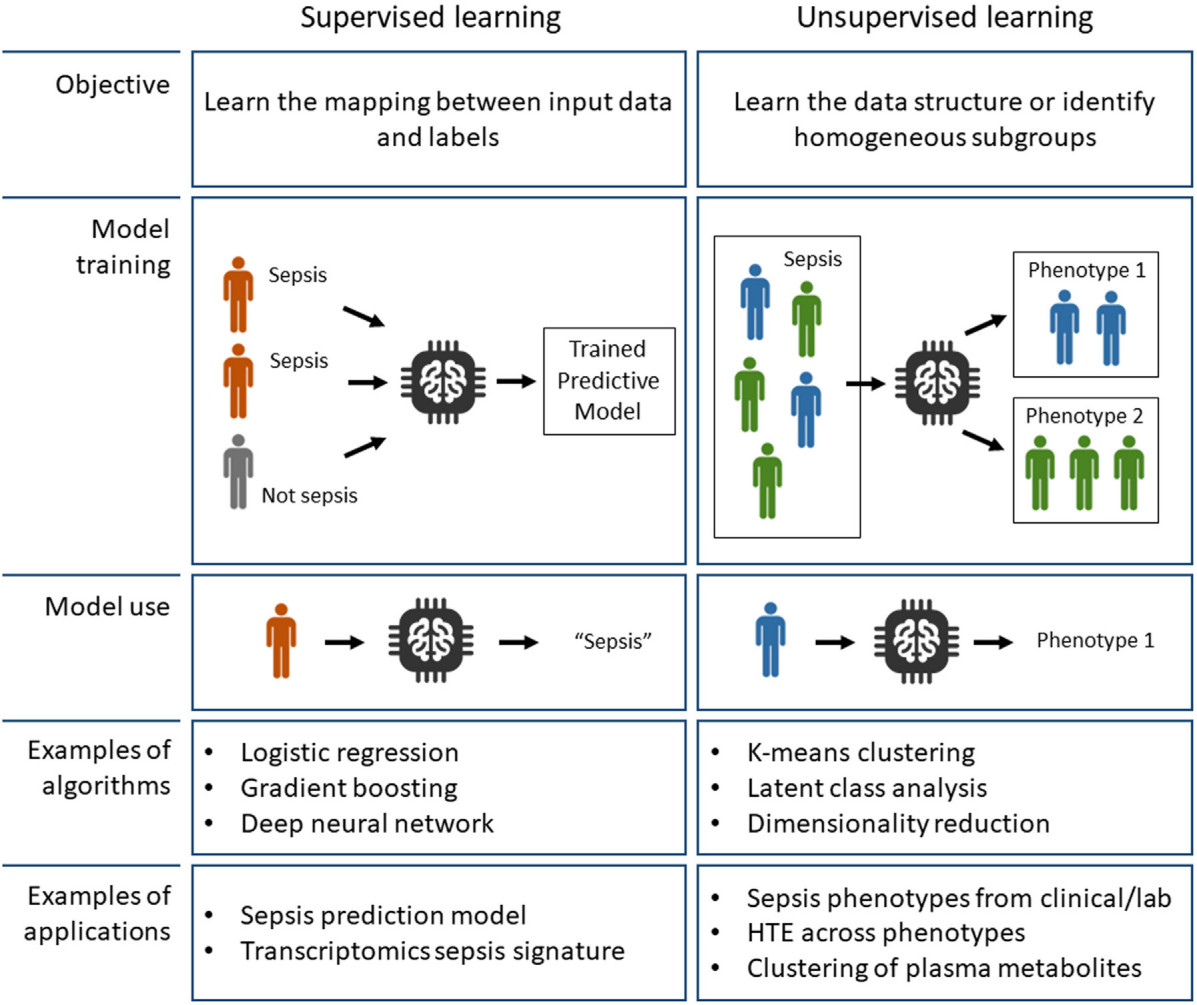
Objectives and principles of supervised and unsupervised learning. Supervised learning methods link input data and labels, and are typically used in sepsis prediction algorithms. Unsupervised learning has been applied to highlight the underlying structure or to unearth hidden patterns in high-dimensional datasets. HTE: Heterogeneity of Treatment Effect.

A variety of machine learning algorithms have been applied to the question of sepsis diagnosis, prognostication and phenotyping, most of which belong to the realms of supervised or unsupervised learning (Fig. 1).

Classically, supervised learning is interested in learning the mathematical function linking some input data (e.g. patient characteristics and severity at the time of hospital admission) and a label (presence of sepsis). As such, supervised learning is applied to prediction tasks, where a model is built on training data and applied prospectively to new -previously unseen-data points. There is a wide range of techniques available to researchers, the most common being logistic regression, decision trees (and their combination: random forests), neural networks (and their “deep” version in deep learning), gradient boosting, among many others. Because they have been used in metabolomics analyses, we also mention Partial Least Squares (PLS), a class of algorithms used to build interpretable predictive models.

Another field of machine learning is represented by unsupervised learning, where the purpose of the algorithm is to establish the underlying structure or hidden patterns in a high-dimensional dataset. Sepsis being a heterogeneous syndrome, the identification of homogeneous phenotypes may allow more targeted therapy, and/or to inform the inclusion of patients into clinical trials.^9^^,^^10^ Here again, a large variety of methods is available to researchers, each with their own advantages and limitations. We'll present here only the most popular algorithms.

Principal component analysis (PCA) is a technique for reducing the dimensionality of large datasets, increasing interpretability (for example allowing their visualisation in 2D or 3D) while at the same time minimising information loss. It does so by creating new, less correlated variables that maximise variance.^11^ t-distributed Stochastic Neighbour Embedding (t-SNE) is another dimensionality reduction technique that can deal with linearly non separable data, mainly used for visualisation of data in 2D and 3D. K-means clustering is one of the simplest and popular unsupervised machine learning algorithms. In k-means, the number of cluster centroids k needs to be pre-defined. Then, the algorithm identifies the location of these centroids and allocates all data points so as to minimise the distance within the clusters. Hierarchical cluster analysis (HCA) is a strategy that seeks to build a hierarchy of clusters that has an established ordering from bottom to top. In HCA, it is not necessary to pre-set the number of clusters. Instead, researchers choose this number of clusters, using a variety of tools such as elbow plots or the dendrogram. Finally, latent class analysis (LCA) and latent profile analysis (LPA) are techniques that aim to recover hidden groups from observed data. They are similar to clustering techniques but more flexible because they are based on an explicit model of the data, and account for uncertainty in the group definition. LCA and LPA are useful when you want to reduce a large number of continuous (LPA) or categorical (LCA) variables to a few subgroups. There are longitudinal versions of both LPA and LCA, which can be used to group time series of patient data, i.e. “trajectories”.^12^

## Sepsis biomarkers and diagnostic tools

Our main results are summarised in Table 1.

**Table 1:** Summary of main findings, with examples of applications.

Task → Input Data ↓	Sepsis diagnosis and/or prediction	Sepsis phenotypes (characterisation of subgroups)	Sepsis prognosis	Treatment decision support
Routine clinical data & lab tests	Sepsis/septic shock prediction tool^13^	Seymour's α, β, γ, and δ^10^	Sepsis mortality prediction^14^	Haemodynamic phenotypes^15^; Coagulation phenotypes and thrombomodulin^16^
Gene expression - transcriptomics	IMX-BVN-1^17^	SRS 1-2^18^^,^^19^; Mars 1-4^20^; Inflammopathic, Adaptive, and Coagulopathic phenotypes^21^	IMX-SEV-2^22^	Interaction SRS-steroids^23^
Inflammatory biomarkers	Presepsin, procalcitonine, pro-adrenomedullin, adrenomedullin^24^	ARDS hypo/hyper inflammatory phenotypes^25^, ^26^, ^27^, ^28^	TNFα levels correlate with mortality^29^	Interaction ARDS phenotypes with PEEP,^25^ fluid strategy,^27^ simvastatin^26^; IFNγ/IL10 ratio and steroids.^30^
Metabolomics	Metabolomic profile of sepsis^31^, ^32^, ^33^	Clustering of plasma metabolic profiles^34^	Prediction of sepsis mortality from blood metabolites^35^^,^^36^	

We classified sepsis biomarkers and digital signatures based on the input data and the objective of the task (sepsis diagnosis, prediction, prognostication, phenotyping or treatment decision support). SRS: Sepsis Response Signature; IMX-BVN-1: Inflammatix Bacterial Viral Non-Infected version 1; IMX-SEV-2: Inflammatix Severity version 2; TNFα: Tumour necrosis factor α.

### Sepsis diagnosis and prediction using machine learning

#### Routine clinical data and laboratory tests

The literature is replete with examples of sepsis prediction models, that use routine clinical data to attempt to identify patients at high risk of sepsis and septic shock. For example, Mao and colleagues derived *InSight*, a sepsis/septic shock prediction tool, in a multicentre cohort of 684,000+ patients, that relied only on 6 vital signs.^13^ For the detection of sepsis and severe sepsis (sepsis with organ dysfunction), their algorithm achieved an area under the receiver operating characteristic (AUROC) of 0.92 (95% CI 0.90–0.93) and 0.87 (95% CI 0.86–0.88), respectively. Four hours before onset, it predicted septic shock with an AUROC of 0.96 (95% CI 0.94–0.98) and severe sepsis with an AUROC of 0.85 (95% CI 0.79–0.91).

A 2020 meta-analysis by Fleuren and colleagues identified 28 papers reporting on 130 various models.^37^ For the prediction of sepsis, diagnostic test accuracy assessed by the AUROC ranged from 0.68 to 0.99 in the ICU, to 0.96–0.98 in-hospital and 0.87 to 0.97 in the ED.

In an evolution of these supervised learning methods, COMPOSER (COnformal Multidimensional Prediction Of SEpsis Risk) was proposed.^38^ It is a deep learning model for the early prediction of sepsis, specifically designed to reduce false alarms by detecting unfamiliar patients/situations arising from erroneous data, missingness, distributional shift and data drifts. Using 40 clinical variables (6 static demographic and 34 dynamic vital signs and lab values), COMPOSER achieved high AUROCs ranging from 0.92 to 0.95 in the ICU and 0.94–0.95 in the ED.

Sporadically, some of these models have been deployed in the clinical environment and tested for their effect on sepsis recognition and patient outcomes. Recently, a large prospective multicentre study monitored over 590,000 in-hospital patients and flagged 6877 patients with sepsis who were identified by an alert before initiation of antibiotics.^39^ The underlying algorithm used Cox proportional hazard models and a wide range of patient data including vital signs, laboratory data, clinician notes, procedures and medication history to generate a sepsis risk score in real time. In the study, patients whose alert was confirmed by a provider within 3 h of the alert had a reduced in-hospital mortality rate (3.3% of adjusted absolute reduction, confidence interval (CI) 1.7–5.1%, and 18.7% of adjusted relative reduction, CI 9.4–27.0%), organ failure and length of stay.

With regards to sepsis prognostication, many publications performed mortality prediction tasks focusing on cohorts of patients with sepsis. For example, van Doorn and colleagues used gradient boosting to predict 31-day mortality in patients with sepsis in the emergency department, using a combination of clinical and laboratory data.^14^ Their model achieved an AUROC of 0.84 (95% CI: 0.81–0.87), surpassing human clinicians (AUROC 0.73) and risk scores (SOFA AUROC 0.75).

#### Non routine biomarkers: transcriptomics, proteomics and metabolomics

A large number of studies have attempted to identify sepsis signatures in omics data collected in the host, but very few were validated prospectively. For example, transcriptomic data (29 mRNAs) of 1,069 patients from 18 retrospective studies was used to build neural network classifiers for bacterial and viral infections.^17^ When tested prospectively in an independent cohort of patients within 36 h of hospital admission, the model achieved an AUROC of 0.92 for bacterial (versus others) infections and 0.91 for viral (vs others) infections. Sampson and colleagues prospectively tested two proprietary blood transcriptomic signatures of bacterial and viral infection.^40^ Here again they conducted a prospective validation study in a cohort of 332 patients with fever in the emergency department, and measured an AUROC of 0.95 (95% CI 0.9–1) using a score combining both transcriptomic signatures.

Risk stratification and mortality prediction in sepsis can be enriched using transcriptomic data. For example, Sweeney and colleagues created four prediction models using data from 21 different sepsis cohorts (both community-acquired and hospital-acquired, N = 1113 patients), which achieved AUROCs around 0.85.^41^ Other work has confirmed the ability of transcriptomic approaches to enhance sepsis mortality prediction in the emergency department^22^ and in surgical populations.^42^

A large number of inflammatory biomarkers (C-reactive protein, presepsin, procalcitonine, proadrenomedullin, adrenomedullin, kallistatin, etc.) have been studied and were found to be associated with the presence of sepsis, with sepsis severity and sepsis outcomes.^24^^,^^43^^,^^44^ For example, a meta-analysis of 19 studies including a total of 3012 patients revealed AUROCs of 0.84 for procalcitonin and 0.87 for presepsin for the diagnosis of sepsis.^24^

Metabolomics is an omics science that uses techniques such as nuclear magnetic resonance (NMR), spectroscopy or mass spectrometry to measure a wide range of metabolites in biofluid samples. This approach has been used in many sepsis studies, utilising a range of supervised learning techniques such as partial least squares discriminant analysis (PLS-DA) and orthogonal projections to latent structures discriminant analysis (OPLS-DA).^45^ In brief such techniques have provided insight into the metabolic disturbance of sepsis and have demonstrated that similar metabolic pathways are disrupted when patients with sepsis are compared to non-septic inflammation as when sepsis survivors are compared with those who die. Sepsis is metabolically characterised by mitochondrial dysfunction and upregulation of glycolysis and the TCA cycle whilst increased energy demand and oxidative stress lead to changes in protein and amino acid metabolism. Many of these studies have been designed to look for metabolites that discriminate disease entities, such as differentiating sepsis from SIRS^31^, ^32^, ^33^ or healthy controls^46^^,^^47^ or sepsis survivors from non-survivors.^35^^,^^36^^,^^45^ For example, Kosyakovsky and colleagues measured the levels of 411 plasma metabolites in 60 patients with sepsis and highlighted 13 molecules that were strongly associated with 28-day mortality, through an ensemble machine learning importance score.^36^

Detrimental sepsis effects have been attributed to a “cytokine storm.” However, a recent meta-analysis found no association between TNFα levels and sepsis source, sepsis severity, or sequential organ failure assessment score, even though TNFα levels were higher in non-survivors.^29^ Plasma metabolites (especially those in the death-related metabolic pathways - DRMPs) differ between sepsis survivors and non-survivors. In a meta-analysis of 21 sepsis cohorts and 2509 metabolites, prediction of death using DRMPs yielded a pooled AUROC of 0.81 (95% CI 0.76–0.87).^48^ In a prospective validation study, the DRMPs metabolites achieved an AUROC of 0.88.

Finally, we briefly mention a potential novel technique that relies on the measurement of the biophysical properties of white blood cells as they are stretched through a microfluidic channel, which accurately identified subjects with severe illness as measured by SOFA, APACHE-II, hospital-free days, and ICU admission.^49^

### Sepsis phenotypes

#### Routine clinical data and laboratory tests

Applying LPA to clinical and laboratory data, Zhang and colleagues defined four sepsis phenotypes.^12^ Profile 1 (the largest) was characterised by the lowest severity and mortality rate; profile 2 was characterised by respiratory dysfunction; profile 3 included multiple organ dysfunction, and profile 4 was characterised by neurological dysfunction. Subsequently, Shald and colleagues examined the progression of these four phenotypes in a different cohort.^50^ As in the seminal work, they confirmed differences in mortality, length-of-stay, ventilator-free days and fluid balance status across the groups.

Seymour et al. conducted a retrospective analysis using routine data from 63,858 patients in three observational cohorts, and applied consensus k-means clustering to characterise four novel sepsis phenotypes (α, β, γ, and δ) with different demographics, laboratory values, and patterns of organ dysfunction.^10^ The α phenotype included patients with the lowest severity. Patients in the β phenotype had more comorbidities and renal dysfunction. Inflammation and pulmonary dysfunction was marked in the γ cluster, while the δ phenotype had more septic shock, liver dysfunction and the highest average SOFA score and mortality rate (32%). The researchers demonstrated that the clusters correlated with biomarkers and mortality. Then, they conducted simulations using data from three randomised clinical trials involving 4737 patients, and demonstrated how the outcomes related to the treatments were sensitive to changes in the distribution of these phenotypes.

Apart from hypothesis generation, the approach of phenotyping entities may find a practical application in sepsis, and in intensive care in general. A potential important contributor to many negative RCTs is our inability to identify subgroups of patients that could benefit from (or be harmed by) a given treatment.^9^^,^^51^ The heterogeneity of patients included in such large trials contributes to ‘negative’ results. Among all patients who meet the rather nonspecific definition of sepsis (or ARDS, delirium, etc.), it is likely that a wide breadth of responses to a given intervention will be seen in different subgroups, ranging from clear benefit to clear harm. Unsupervised learning could help inform future trials by highlighting which subgroups of patients are more likely to respond to a given intervention. For example, clustering has enabled the characterisation of coagulation phenotypes, and demonstrated the association between cluster assignment and response to recombinant human thrombomodulin.^16^ Another study identified various haemodynamic profiles of patients with sepsis (e.g. hypovolaemic, hypervolaemic, left ventricular failure, etc.), all of which require a different management.^15^ However, this approach has not been successfully applied yet in a prospective randomised trial.

Finally, longitudinal clustering has allowed the identification of various patient “trajectories”, describing their progression from disease state to disease state, across the spectrum of severity and organ dysfunctions. For example, Liu has generated four clusters using spectral clustering on time series of patient data, labelled them according to septic shock prevalence within clusters, and successfully linked them to outcomes.^3^

#### Non-routine biomarkers

##### Gene-expression

Machine learning techniques have been applied to gene expression data in an attempt to identify subgroups of patients with sepsis who have different clinical outcomes and may respond differently to sepsis therapies, based on differing pathophysiological mechanisms driven by different patterns of gene expression. Agglomerative hierarchical cluster analysis with k-means clustering has been used to derive two “sepsis response signatures” (SRS) from leucocyte transcriptomics data in patients with either community acquired pneumonia or faecal peritonitis.^18^^,^^19^ Differential gene expression between these two sub-phenotypes suggests that patients with the SRS1 phenotype have gene expression features consistent with relative immunosuppression compared to those with the SRS2 sub-phenotype, characterised by endotoxin tolerance and T-cell exhaustion. The SRS1 subgroup is associated with more organ dysfunction, vasopressor requirement and a higher mortality rate.^18^^,^^19^ Not only do the identification of such sub-phenotypes provide novel pathological insights that could lead to better understanding of sepsis, they provide a means by which we could start to identify groups of patients who may derive the most benefit or may come to harm from sepsis therapies. In a secondary analysis of a randomised trial of corticosteroids in septic shock, a significant interaction between SRS groups and response to steroids was found, with those patients with the SRS2 (relatively immunocompetent) sub-phenotypes having increased mortality if given hydrocortisone compared to placebo.^23^

Sweeney and colleagues pooled data from 14 bacterial sepsis transcriptomic datasets totalling 700 patients and used clustering to identify three phenotypes, which they call Inflammopathic (innate immunity activation), Adaptive (adaptive immune activation), and Coagulopathic (with coagulopathy).^21^ They were able to externally confirm the robustness of these phenotypes in 9 other datasets (n = 600).

In another study, consensus clustering based on iterations of agglomerative hierarchical clustering of genome wide leukocyte gene expression data derived four sub-phenotypes named Mars1-4.^20^ Mars1 was associated with a decrease in expression of genes corresponding to key innate and adaptive immune cell functions such as toll-like receptor, NF-kB1 signalling, antigen presentation, and T-cell receptor signalling and an increase in expression of metabolic pathway genes including haem biosynthesis pathways. This sub-phenotype had high SOFA scores and incidence of shock and had the highest mortality of all of the sub-phenotypes. Alternatively, the relatively low risk Mars3 sub-phenotype had increased expression of adaptive immune and T-cell function and showed a significant association with the SRS2, low risk, sub-phenotype described previously. Interestingly, when applied to an exploratory data set derived from children under 10 years of age with sepsis, although Mars1, 2 and 4 groups could be detected Mars3 was absent, perhaps highlighting the underdeveloped adaptive immune system in children.^52^

An alternative approach using partitioning around medoids (PAM) clustering based on Euclidean distance on gene-expression data from neutrophils of patients with sepsis^53^ also identified two subgroups with one associated with higher rates of severe sepsis and increased expression of genes associated with inflammation and Toll receptor signalling pathways.

In paediatric sepsis, unsupervised hierarchical clustering of 6934 genes revealed three sub-phenotypes named A, B and C.^54^ An analysis of the 100 most predictive genes revealed that sub-phenotype A was characterised by repression of genes corresponding to key signalling pathways of the adaptive immune system. This sub-phenotype was also associated with the worst disease severity, highest rates of organ failure and mortality. Subsequently, gene expression mosaics were developed based on these 100 genes using self-organising maps for every sample,^55^ an approach that demonstrated high accuracy in a sub-phenotype classification task. Mosaics could also be analysed by computer based image analysis platforms which allowed the three subgroups to be validated in a second data set.^56^ As in adults, an association between corticosteroids and mortality was identified in children, with sub-phenotype A associated with higher mortality if given corticosteroids.^57^

Recently, prospective validation of such mRNA arrays has been conducted. For example, IMX-SEV-2 is a 29-host-mRNA classifier designed to predict disease severity in patients with acute infection or sepsis.^22^ For predicting in-hospital mortality, IMX-SEV-2 had an AUROC of 0.84 (95% CI: 0.76–0.93), higher than lactate, qSOFA or NEWS2.

##### Inflammatory phenotypes

Another avenue for the use of machine learning techniques has been to look for inflammatory sub-phenotypes based on measurements of panels of inflammatory mediators either alone or in conjunction with clinical variables.

The area in which this has had most success is in Acute Respiratory Distress Syndrome (ARDS), a common complication of sepsis. LCA has been applied to several ARDS trials datasets that consist of both routine variables and inflammatory biomarkers. Across all analyses, two sub-phenotypes have been consistently identified^25^, ^26^, ^27^, ^28^ with a hyper-inflammatory sub-phenotype having features such as higher levels of IL-6, IL-8, sTNFR1, higher rates of vasopressor use and lower circulating protein C and bicarbonate than a hypo-inflammatory sub-phenotype. Importantly in reanalysis of clinical trial data these inflammatory sub-phenotypes have been found to have differential responses to several trial therapies including high or low PEEP strategy,^25^ liberal versus conservative fluids^27^ and simvastatin.^26^ In a drive to turn these sub-phenotypes, that require large panels of data and complex clustering methodologies, into entities that could be used with fewer variables in the clinical environment, feature selection models have been applied. These derived parsimonious models,^58^ and highlighted bicarbonate, IL-6, IL-8, protein C, sTNFR-1, and vasopressor use as the most predictive variables.

In another re-analysis of data coming from a randomised trial, machine learning identified the IFNγ/IL10 ratio to be a good biomarker for the decision to administer hydrocortisone in septic shock.^30^ This pattern was confirmed in three separate sepsis trials data.

A similar approach has since been taken in COVID-19^59^ where there was a significant overlap between COVID-19 latent classes and ARDS inflammatory phenotypes and where response to corticosteroids was seen to differ between the two derived classes. There is now increasing evidence that such inflammatory sub-phenotypes may be seen in other critically ill populations, for example, pancreatitis^60^ and ventilated patients without ARDS.^61^

Hierarchical clustering has been applied to panels of cytokines, chemokines and growth factors in sepsis^62^, ^63^, ^64^, ^65^ with two to three clusters being identified depending on the panels of mediators used and the types of patients included (sepsis, SIRS and healthy controls). As such it is difficult to draw parallels between these studies. However, in the largest of these^62^ three clusters with high, medium and low cytokine concentrations were identified. Patients in the high cluster had higher rates of shock, more coagulation failure and a higher mortality. However, these studies are generally small and, as yet, not validated making their clinical relevance uncertain.

##### Metabolic phenotypes

Clustering approaches to find novel subgroups of patients with sepsis have rarely been applied to metabolic datasets. In a rare example, hierarchical clustering of plasma metabolic profiles revealed three clusters with metabolic differences between groups being mainly driven by plasma lipids.^34^ The first group was associated with the highest mortality and rates of septic shock and had reduced levels of lipids compared to group 3, the lowest mortality group. The lipids differentiating groups were mainly fatty acid metabolites, lysophospholipids and sphingolipids.

## Discussion

This narrative review highlighted recent developments in the application of machine learning models for sepsis identification and/or phenotyping, towards a more accurate characterisation of its heterogeneous clinical pictures. Novel models in use, and under development, which rely on patient vital signs along with routine clinical and laboratory data, have yielded promising results for early sepsis detection and prediction.^37^^,^^39^ Evaluation on non-routine datasets, to establish gene expression, inflammatory and metabolomic sepsis phenotypes have deepened such analyses further, crucially informing us on the use of therapies such as steroids.^23^^,^^30^^,^^57^^,^^59^ The research highlighted here offers a number of potential future applications: these tools, in conjunction with routine clinical and biological information, may improve timeliness and accuracy of diagnosis, suggest physiological pathways and therapeutic targets, inform targeted recruitment into clinical trials, and optimise clinical management. The potential for machine learning to better target the immune response in sepsis through precision medicine and enrichment is promising, after well over 100 “traditional” clinical trials were conducted targeting the systemic inflammatory response, without significant positive results.^51^^,^^52^

However, there remain significant limitations and work ahead before these tools realise their full potential, are validated at the bedside, become certified medical devices and/or inform sepsis trials inclusion.

Firstly, the challenge of correct sepsis labelling remains pressing. Wide discrepancies in sepsis cohorts are identified when different sepsis definitions and identifications methods are applied.^66^ Bedside screening tools to detect these patients, such as the quick Sequential Organ Failure Assessment (qSOFA), lack sensitivity.^67^ Physicians themselves often cannot agree on the presence of an infection.^68^ Some patients who are initially not even categorised as having sepsis might benefit greatly from early administration of antibiotics.^69^ If inaccuracies or biases are present in training datasets, supervised machine learning models (used in sepsis prediction models) could perpetuate those errors or prejudices. However, machines may be beneficial in instances where, for example, the amount of data to process or the pace at which it is generated becomes larger than what a human can handle.

Secondly, the vast majority of these models have received little to no prospective validation, so their external validity remains unknown. For example, Fleuren and colleagues in 2020 identified only three prospective studies and one randomised trial (with 142 patients) on sepsis prediction in the entire literature.^37^ A common challenge in developing these sepsis biomarkers or predictive models is the risk of overfitting: that the results apply solely to the data in which a biomarker was developed (or a model was trained), and fail to generalise to external cohorts.^70^^,^^71^ For example, this pitfall can be illustrated by the incomplete overlap between the transcriptomics immunosuppressed phenotypes SRS-1 and Mars-1,^70^ or by the poor performance of some widely deployed sepsis prediction models.^71^^,^^72^ In the field of transcriptomics, an important contribution to this issue has been in studies pooling gene expression data from multiple cohorts (sometimes upwards of 30) in attempts to identify global sepsis signatures, shared across populations.^41^^,^^70^ This comes with unique challenges such as correcting for the “batch effect”, since gene expression levels are dependent on the patients’ clinical and ethnic background, as well as on the platform used for profiling. Methods have been developed to make datasets comparable, such as co-normalisation algorithms, which allows to “align” gene expression levels using the values measured in healthy controls in the studies.^41^ Several teams demonstrated some success in the external validity of the signatures developed,^21^^,^^41^^,^^42^^,^^70^ as well as in a few prospective observational studies.^22^

A third limitation is that the vast majority of the research is still limited to high-income western countries, although about 85% of sepsis cases in the world occur in low and middle income countries.^73^

Finally, one could argue that the use of non-routinely collected data (gene expression, cytokines …) limits for the foreseeable future the opportunity to use these technologies to actually inform clinical practice and trial enrolment, unless researchers manage to identify a sparse set of biomarkers, or even better, a unique biomarker.

In conclusion, machine learning, in conjunction with routine clinical and biological data, has potential in precision medicine and enrichment in adult and paediatric sepsis. However, the bulk of the published applications is represented by early, proof-of-concept research that remains to be tested for patient benefit.

## Outstanding questions

Most of the literature on machine learning models and biomarkers in sepsis remains limited to small cohorts and retrospective analyses.

How will the research community interact with the industry to translate these academic projects into certified and validated medical devices?

What factors will determine which of those tools will become widely available and used at the bedside?

Which of these applications, if any, will demonstrate wide scale and consistent clinical utility, positive effects on patient outcomes and/or healthcare delivery?

## Search strategy and selection criteria

Data for this review were identified by searches of MEDLINE, PubMed, Google Scholar and references from relevant articles using the search terms “sepsis”, “machine learning”, and “biomarkers”. Only articles published in English between January 2000 and October 2022 were included.

## Contributors

MK: conceptualisation, methodology, writing (original draft, review and editing); AG: writing (original draft, review, and editing); KT: writing (original draft, review, and editing); CS: conceptualisation, methodology, writing (original draft, review, and editing); DA: conceptualisation, methodology, writing (original draft, review, and editing). All authors read and approved the final version of the manuscript.

## Declaration of interests

MK: consulting fees (Philips Healthcare), speaker honoraria (GE Healthcare). CS: consulting fees (Beckman Coulter and Inotrem), personal fees for data safety monitoring board or advisory board (RENOVATE trial) and AE role at JAMA.
